# Assessment of Knowledge and Attitudes on Genetically Modified Foods Among Students Studying Life Sciences

**DOI:** 10.7759/cureus.32744

**Published:** 2022-12-20

**Authors:** Devanshi Rathod, Radhika P Hedaoo

**Affiliations:** 1 Nutrition, Symbiosis Institute of Health Sciences, Pune, IND

**Keywords:** genetically modified foods, life science curriculum, genetically modified organism, life science, genetically modified products

## Abstract

Background: Genetic engineering has stimulated interest in a range of fields, including agribusiness, food technology, food product development, and nutrition. Even though the public opinion on genetically modified (GM) goods is polarizing, the majority of experts believe that the advantages outweigh any potential risks, if at all there are any. As a result, the role of science education is to prepare students to be citizens who have a fundamental understanding of genetic engineering. As the students of life sciences are the future scientific experts and scholars who progress in the subject of genetically modified organisms (GMOs), they need to have correct knowledge of GMOs, GM foods, and appropriate attitudes regarding the same.

Methodology: To investigate the knowledge and attitudes of life-sciences university students (n= 203) concerning GM foods, a cross-sectional observational survey-based study was carried out by administering a structured questionnaire across three disciplines of life sciences - Biotechnology, Food Technology, and Nutrition for undergraduates, postgraduates, and post-graduate diploma students. The scores for knowledge and attitudes were divided into tertiles as high, moderate, and low scores.

Results: 88.2% of the participants agreed to have read about GMOs in their curriculum and 76.8% had defined GM foods correctly. When the participants were categorized into tertiles, it was observed that out of all the high scorers, 45.5% were food technology majors and 43% were biotechnology majors and only 11.3% were nutrition majors. 63.1% of students were found to be in favor of GMOs and GM foods and had a positive attitude toward them. There was a moderately positive association of knowledge levels with attitudes toward GMOs and GM foods (p< 0.05).

Conclusion: Although in general, the life-sciences students had the basic knowledge of GMOs and GM foods, the food technology, and biotechnology majors had better knowledge about GMOs and GM foods as compared to nutrition majors. The attitude scores were directly proportional to knowledge scores which emphasizes the need for robust science education on comprehending the topic better. Incorporating a GM-related curriculum for nutrition discipline can help students learn better about the issues surrounding transgenic technology, food safety, and nutrition.

## Introduction

Over the last few years, genetic engineering has stimulated interest in a range of fields, including agribusiness, food technology, food product development, and nutrition. Genetic technology has emerged as a very debatable topic in recent times as there are several studies available regarding it and there is both positive and negative outlook to it. 

The United Nations (UN) Cartagena Protocol on Biosafety defines a "living modified organism (LMO) as a genetically modified organism (GMO)". If a plant fits two criteria, it is considered genetically modified (GM): One, the plant includes a unique genetic material combination; second, the modified genetic material was introduced by modern biotechnology. Raman stated that "Modern biotechnology is defined as the use of in vitro nucleic acid procedures (such as recombinant DNA and direct nucleic acid injection into cells or organelles) or the fusing of cells from different taxonomic families [1]. In genetic engineering, there are specific changes that are made in the genetic makeup such as addition, deletion, or the alteration of the base pairs that code for certain characteristics to attain discrete changes in the organism. The major objective of GM crops is to append novel attributes to the crops that were not present in it naturally. Potential advantages of GM foods are reducing world hunger, better nutritional value, food development with specific nutritional objectives, development of more effective crops that use nitrogen and other nutrients is ongoing, reduction in the use of pesticides and herbicides, minimizing the use of herbicides proves better for the environment and also decreases the cost of the farmers, production of improved quality crops as suggested by Turnbull et al. [2].

One of the advantages of GM crops developed today is the enhancement in the nutritional traits of certain crops. In order to address world nutrition problems like vitamin deficiencies, food allergies, hidden hunger, and food security, a variety of “nutritionally enhanced” GM crops are in process of development [2,3].

In India, GM technology in crops productions and food has been introduced comparatively late but has gained acceleration in recent times. Only in 2002, India commenced with the commercial cultivation of its first GM crop, Bt Cotton. Furthermore, India has made remarkable progress in the manufacturing of GM cotton since then. According to Chimata and Bharti, India is now the world's fourth-largest land farmer of GM crops and the world's second-largest cotton producer [4]. Lauterbach and Bantle stated while talking about the regulations and approval of GM crops, the Genetic engineering approval committee (GEAC) is regarded as the main constituent body [5].

There have been studies regarding consumers’ knowledge and attitude with respect to GM crops and foods. These studies took the layman and the population at large into consideration and found that, due to a lack of adequate information and education, society had a negative perception regarding genetic technology as observed by Deodhar et al. [6]. Thus, correct information and education play a very important role in creating an informed and positive perception of GM foods. Chen et al. also suggest that this enables consumers to make an informed choice about their intake and consumption of these processed foods [7]. The youth demographic is the most vulnerable to the health risks connected with GM foods.

Therefore, the role of science education is to prepare students to be citizens who have a fundamental understanding of genetic engineering as suggested by Aerni [8]. A few research studies on the student perceptions and knowledge of GM foods including Montuori et al. have led to the conclusion that GM food consumption is mostly influenced by knowledge and its impact on human health [9]. Students and millennials who are educated and knowledgeable about biotechnology and specific sciences perceive less risk with respect to GM food products as stated by Oz et al. [10]. The current students of life-sciences majors are potential future experts in the industries of agriculture, biotechnology, and food technology and are most likely to be involved in discussions and panels on GMOs appropriately stated by Wnuk and Kozak [11]. Emerging adults' awareness of GM foods will enable them to actively participate in conversations about safety precautions for using GM foods. Hence the present study explored the knowledge, attitudes, and practices of students regarding GMOs as there is a difference between the knowledge related to GMOs and their practices across various disciplines of life sciences.

## Materials and methods

The study was designed as a cross-sectional, observational study. A total of 203 students were included in the study. The students from three different majors of life sciences i.e., Nutrition, Biotechnology, and Food-technology enrolled in either BSc, MSc, or Post-graduate Diploma management (PG-DM) programs were selected by purposive-convenience sampling and recruited online by sending google forms for consent to participate in the study through students online forums, Facebook, WhatsApp groups, and Linkedin. Students from other courses and specializations were excluded from the study. Ethical approval for the study design and questionnaire was obtained from the Independent Ethics Committee (SIU/IEC/396) of Symbiosis International University, Pune India. Informed consent from each participant was obtained before conducting the survey. A structured questionnaire was formulated after reviewing the relevant literature [12-14] and the developed questionnaire was pretested (n=10) and was checked for content validity with the help of an expert from the field of genetics. The scores obtained were categorized into tertiles as high, medium, and low scores on the basis of the responses obtained.

Statistical analysis was carried out using SPSS, version 28.0.1 (IBM Corp., Armonk, NY). Numerical, percentage and mean values in tabular and graphical representations were used to interpret the data. The chi-square test was used to compare the different courses of life sciences and their knowledge about GMOs as well as differences in attitudes toward GM foods among the three majors. One-way ANOVA posthoc Tukey’s was used to analyze the association between the level of knowledge of the students and their attitude toward GM foods. Pearson’s correlation coefficient was used to analyze the association between the knowledge and attitudes of the students regarding GMOs and GM foods. P-value < 0.05 was considered significant.

## Results

**Figure 1:**
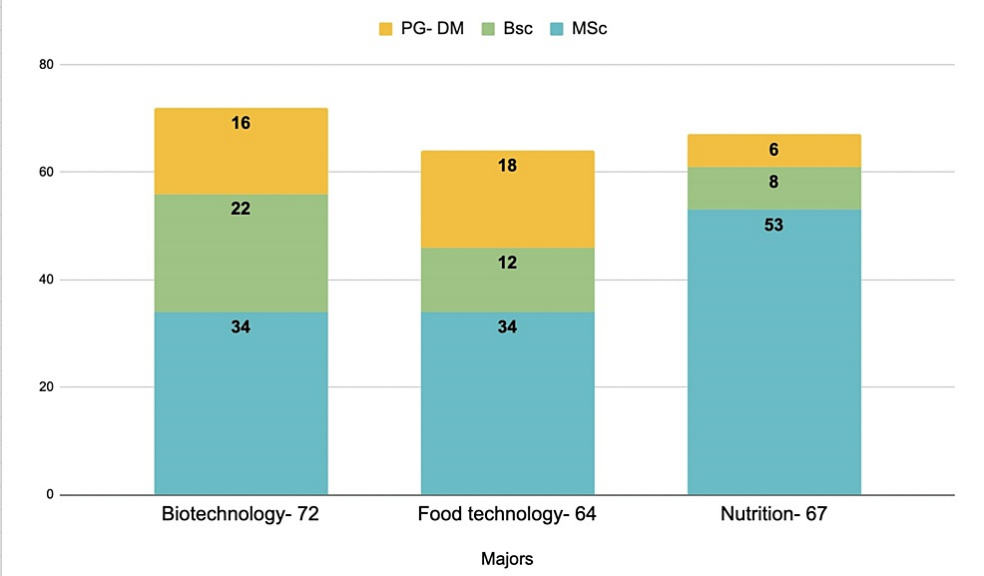
Number of participants under different majors M.Sc- Masters of Science; B.Sc- Bachelors of Science; PG-DM- Post-Graduate Diploma

Understanding the concepts of GMOs involves interdisciplinary knowledge from a variety of domains, including biology, chemistry, and genetics. Students under different specializations have varied levels of knowledge. A total sample of 203 students from different life-sciences disciplines was included in the study. The distribution of participants under different majors and education levels is given briefly in Figure 1. 

The maximum number of participants i.e., 35.5% belonged to biotechnology majors followed by nutrition (33%) and food technology (31.5%) and most of them were master’s students (59.6%). When the participants were asked about awareness of GMOs, 88.2% of the participants agreed to have heard about GMOs and most of them (76.8%) had defined GM foods correctly (Table 1). However, there were significant differences found in the responses of the students across the three majors regarding the production year of the first-ever GM crop and their opinion on the effects of GM foods on the environment. It was observed that predominantly biotechnology and food technology students had correctly answered the questions “When was the first genetically modified organism (GMO) produced?” and “What are your thoughts on how GM foods/crops affect the environment?” Most students from nutrition answered them incorrectly, i.e., 18.7% and 17.7%, respectively.

**Table 1:** Genetically modified-food related knowledge across different majors of life-sciences (n= 203) Note: Data was analyzed using chi- square test. P-value < 0.05 was considered significant. The data is represented as n(%).

Knowledge related questions regarding GMOs & GM foods	Majors n (%)			P-value
	Nutrition	Food technology	Biotechnology	
Heard about GM organisms				
Correct answer	58 (29)	55 (27 )	66 (33)	0.518
Incorrect answer	9 (4)	9 (4)	6 (3)	
Definition of GM foods				
Correct answer	48 (24 )	49 (24)	59 (29)	0.354
Incorrect answer	19 (9 )	15 (7 )	13 (6)	
Organisms responsible for obtaining genes for GMO/ crops				
Correct answer	47 (23)	49 (24)	47 (23)	0.354
Incorrect answer	20 (10)	15 (7 )	25 (12)	
Traditional breeding and genetic modification similarity				
Correct answer	52 (26)	51 (25 )	50 (25)	0.335
Incorrect answer	15 (7)	13 (6 )	22 (11)	
Production of first GMO- year				
Correct answer	29 (14 )	43 (21 )	44 (22)	0.015*
Incorrect answer	38 (19)	21 (10)	28 (14)	
Benefits of GM foods and crops				
Correct answer	59 (29)	47 (23)	55 (27)	0.089
Incorrect answer	8 (4)	17 (8)	17 (8)	
Effect of GM foods on the human genes				
Correct answer	37 (18 )	50 (25)	45 (22)	0.02
Incorrect answer	30 (18)	14 (7)	27 (13)	
Effect of GM foods on environment				
Correct answer	31 (15)	54 (27)	52 (26)	<0.001*
Incorrect answer	36 (18)	10 (5)	20 (10)	
Effect of GM foods on consumer health				
Correct answer	23 (11 )	50 (25)	48 (24)	<0.001*
Incorrect answer	44 (22)	14 (7)	24 (12)	
Assessment of safety of GM foods before its launch in market				
Correct answer	44 (22)	33 (16)	32 (16)	0.04*
Incorrect answer	23 (11 )	31 (15 )	40 (20)	
Factors determining value of GM foods				
Correct answer	33 (16)	41 (20)	46 (23)	0.134
Incorrect answer	34 (17)	23 (11 )	26 (13)	
Perception and belief of higher nutritive value of GMOs				
Correct answer	46 (23)	58 (29)	63 (31)	0.002*
Incorrect answer	21 (10)	6 (3)	9 (4)	
Main GM crop grown in India				
Correct answer	39 (19)	47 (23)	54 (27)	0.066
Incorrect answer	28 (14)	17 (8)	18 (9)	
Availability of GM foods in Indian market				
Correct answer	38 (19)	55 (27)	51 (25)	0.001*
Incorrect answer	29 (14)	9 (4 )	21 (10)	
Availability of information about GM foods among consumers				
Correct answer	50 (25)	59 (29)	64 (32)	0.01*
Incorrect answer	17 (8.4)	5 (2.5)	8 (3.9)	
Internationally agreed policy on labelling of GM foods				
Correct answer	40 (19.7)	59 (29.1)	61 (30)	<0.001*
Incorrect answer	27 (13.3)	5 (2.5)	11 (54)	

**Figure 2:**
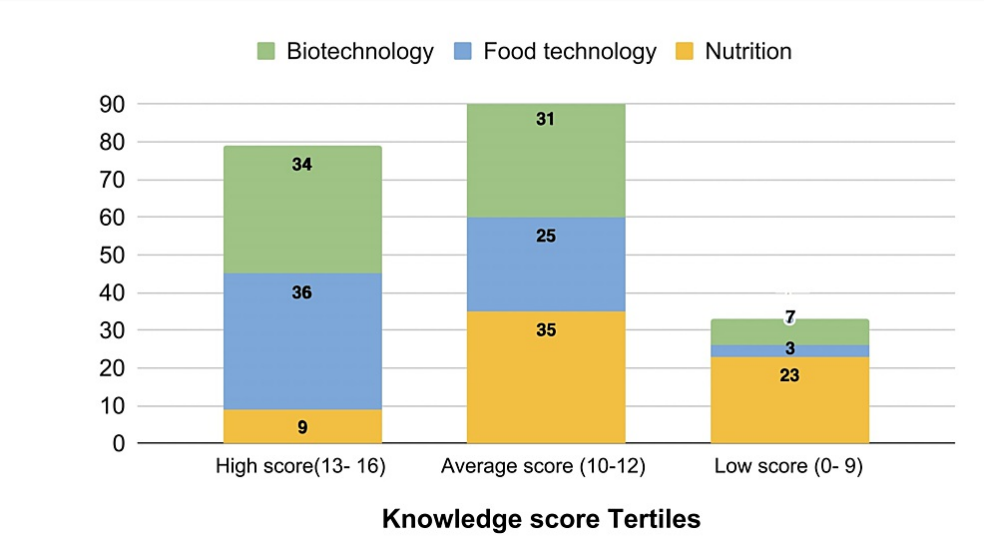
Comparison of total knowledge score in tertiles among the majors (n= 203)

Comparison based on total knowledge score tertiles among the different majors is represented in Figure 2.

Among the high scorers (between 13 to 16), It was observed that 45.5% (n=36) belonged to the food technology majors and 43% (n=34) were from the biotechnology majors. Whereas, nutrition students were among the majority that had average scores followed by biotechnology students. Similarly, the maximum number of students scoring poorly were from nutrition majors.

While comparing the attitudes of the students towards GMOs and GM foods across the majors, a statistically significant difference was found between the majors concerning the distribution of responses to the students’ propositions of “there is very little awareness among the common public regarding GM foods” and “I think product labels should contain information about GMO content of the products” (Table 2). 

**Table 2:** Attitudes towards GM-foods across majors of life-sciences (n= 203) Note: Data was analyzed using the chi-square test. P-value < 0.05 was considered significant. The data is represented as n(%).

Statements about GMOs	Majors n (%)			P-value
	Nutrition	Food technology	Biotechnology	
Support the use of GM foods in India as consumer				
Agree	42 (21)	46 (23)	58 (29)	0.092
Neutral	21 (10)	10 (5)	10 (5)	
Disagree	4 (2)	8 (4)	4 (2)	
Willingness to buy and consume GM foods available in the market.				
Agree	41 (20)	52 (26)	59 (29)	0.132
Neutral	17 (8)	8 (4)	10 (5)	
Disagree	9 (4)	4 (2)	3 (2)	
Endorsements and recommendations to use GM foods.				
Agree	32 (16)	36 (18)	51 (25)	0.087
Neutral	26 (13)	21 (10)	19 (9)	
Disagree	9 (4)	7 (3)	2 (1)	
If I had a farm, I would promote the growing of GM crops.				
Agree	39 (19)	38 (19)	47 (23)	0.391
Neutral	17 (8)	15 (7)	17 (8)	
Disagree	11 (5)	11 (5)	8 (4)	
Perception of awareness among the common public regarding GM foods.				
Agree	51 (25)	40 (20)	51 (25)	0.05*
Neutral	11 (5)	18 (9)	13 (6)	
Disagree	5 (2)	6 (3)	8 (4)	
Support modification of the genetic materials of foods to prolong their shelf life and to produce products more resistant to pests and herbicides.				
Agree	38 (19)	34 (17)	51 (25 )	0.222
Neutral	20 (10)	15 (7)	14 (7)	
Disagree	9 (4)	15 (7)	7 (3)	
Support modification of the genetic materials of foods to enrich their nutritional content.				
Agree	46 (23)	43 (21)	57 (28)	0.21
Neutral	16 (8)	15 (7)	8 (4)	
Disagree	5 (2)	6 (3)	7 (3)	
I think product labels should contain information about GMO content of the products.				
Agree	48 (24)	52 (15)	69 (34)	<0.001*
Neutral	14 (7)	6 (3)	2 (1)	
Disagree	5 (2)	6 (3)	1 (1)	

**Figure 3:**
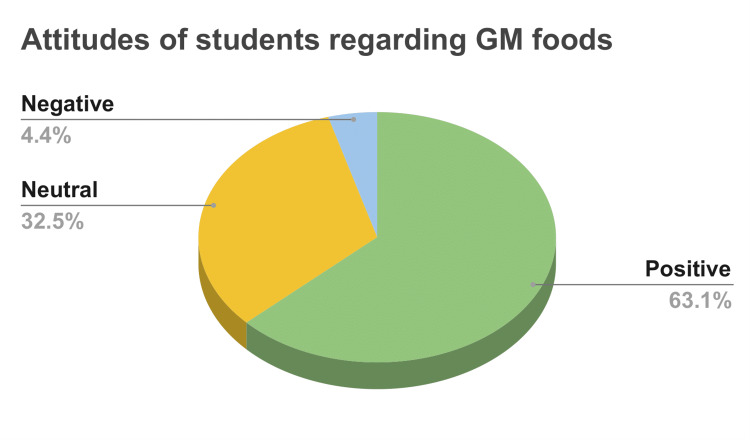
Distribution of the participants according to the attitudes (n=203)

When the participants were classified on the basis of total attitude scores into three types; positive, indifferent, and negative attitude, maximum students i.e. 63.1% were found to be in favor of GMOs and GM foods and had a positive attitude towards it. However, 35.2% had an indifferent attitude and 4.4% still believed GM foods are a threat to the society and environment (Figure 3).

The average knowledge score and attitudes score regarding GMOs and GM foods among the students of different majors were 11.7 ± 2 and 30.7 ± 4.5 respectively. Knowledge scores significantly differed between nutrition and food technology majors and nutrition and biotechnology majors (Table 3). Whereas there was no significant difference found between the knowledge scores of food technology and biotechnology majors. Attitude scores showed significant differences among the three majors. It was also observed that attitude scores were directly proportional to knowledge scores indicating a moderately positive association of knowledge levels with attitudes toward GMOs and GM foods.

**Table 3:** Mean scores for knowledge and attitude towards genetically modified organisms of the study participants (n=203) Note: Data was analyzed using one-way ANOVA post hoc Tukey’s and Pearson’s correlation coefficient; P- value < 0.05 was considered statistically significant. The data is represented as mean ± SD.

Average Score	Overall average (n= 203)	Nutrition (n= 67)	Food technology (n=64)	Biotechnology (n= 72)	P- value	Pearson's r
Knowledge score	11.7 ± 2	10.3 ± 2	12.7 ± 1.6	12 ± 1.9	0.000^#^	0.05*
Attitude score	30.7 ± 4.5	30.5 ± 5.2	29.7 ± 4.5	31.9 ± 3.8	0.019^#^	

## Discussion

Genetic modification technology has been extensively used in the field of biotechnology, agriculture, health, and nutrition. Notwithstanding its widespread use, genetic modification technology is considered to be controversial and has become a highly-relevant topic of discussion. Within the scope of the current study, which was carried out to evaluate students from various life-science disciplines' knowledge of and attitudes about GM foods, it was found that 88.2% of the participants were aware of the concept of GMOs. Similar results were recorded by Wnuk and Kozak who assessed the knowledge and attitudes about GMOs of Polish students of life-sciences faculty, and also by Folkerth where the knowledge and opinions of the students regarding GMOs were examined [11,15].

The mean knowledge score was 11.7 ± 2 and the majority of the participants (44.8%) scored average i.e. between 10 to 12 out of 16. The average knowledge score of food technology students was higher than that of nutrition students and almost equal to that of biotechnology students (Table 3). This indicates that compared to students studying food technology and biotechnology, nutrition students have rather less knowledge about GMOs and foods. Similar findings were noted by Lamanauskas and Makarskaitė-Petkevičienė for Lithuanian university students and Prokop et al. for Slovakian students respectively [16,17]. In the present study, when knowledge scores were compared on the basis of type of major; students primarily studying biotechnology and food technology were found to have provided accurate answers to the questions related to the effect of GM foods on consumer health; assessment of GM foods before its launch in the market; perception and belief of the higher nutritive value of GMOs; availability of GM foods in the Indian market; availability of information about GM foods among consumers; and internationally agreed policy on labeling of GM foods. It was observed that the majority of the participants who scored high belonged to biotechnology and food technology majors. Majority of participants that scored poorly belonged to nutrition majors. This revealed that nutrition students lacked accurate information and knowledge with respect to GMOs and GM foods information. One explanation for this might be the absence of a robust curriculum related to GMOs and GM foods and their effect on health since the nutrition major rather focuses on medical nutrition therapy, food science, food composition, and its impact on human health.

When the students’ opinions were taken for different situational statements to assess their attitudes towards GMOs and GM foods; 71.8% of the participants agreed that they support the use of GM crops and GM foods in India. While 15.2% of the students opposed the genetic modification of crops to increase shelf-life and to produce pest and herbicide-resistant crops, 72% of them supported the genetic modification of crops and foods to enrich their nutritional content. Moreover, the majority of them agreed on society at large has very little awareness about GM foods as well as the necessity of information on GMO content on the food product labels. Overall, 63.1% of students had a positive outlook toward GMOs and GM foods which predominantly comprised biotechnology and food technology students. This is probably due to the fact that biotechnology and food technology students gain more knowledge about GMOs and, somewhat learn to favor novel advancements in biotechnology, of which GMOs are a particularly significant example. However, 35.2% of the study participants which comprised of majorly nutrition students had a neutral attitude towards GM foods which can be attributable to a lack of accurate information has resulted in disinterest and an indifferent attitude. Our findings do not support those of Lamanauskas and Makarskaitė-Petkevičienė who found that both biology and non-biology students opposed GMOs and warned of the risks to both humans and the environment [16].

The correlation test between knowledge scores and attitude scores indicated a moderate correlation. It was observed that a more positive attitude came from higher knowledge about GM technology. This result from our study was found to be similar to that of Turker et al. [14] who assessed the knowledge and attitudes toward GMOs among Turkish nursing students, and another study by Oz et al. was carried out on the American population [10]. On the contrary, Oselinsky et al. reported no differences in attitudes toward GMOs according to degrees of education [18]. The current study explored the perspectives of students on GMOs and GM foods studying professional life-science courses who are future leaders, scientists, and significant members who will be involved in GMO-related projects and studies. The detailed curriculum on GMOs and GM foods is crucial to shaping the attitudes and enhancing the knowledge of students.

## Conclusions

It was concluded that although in general, the life-sciences students have fairly good knowledge of GMOs and GM foods, the three different majors of life sciences- nutrition, food technology, and biotechnology had different levels of understanding of GMOs and GM foods. It was observed that their insights into GMOs and perspectives toward them differed, sometimes quite significantly. The majority of the participants had a positive attitude toward GMOs and GM foods. However due inclusion of curriculum related to GM technology, food technology, and biotechnology students were strong respondents i.e. they had a fixed attitude towards GM foods. On the other hand, the majority of students with neutral attitudes belonged to the nutrition majors which concluded that they were indifferent to GMOs. Therefore, incorporating a GM-related curriculum can help students learn more about the issues surrounding GM food and food safety. This will aid students of nutrition majors to create a better outlook toward the development of genetic technology. Knowledge about GMOs and GM foods and attitude towards it are directly related to each other which stipulates that the greater the knowledge, the more positive attitudes towards GMOs and GM foods are to be found. The present study assessed knowledge and attitudes regarding GMOs and GM foods among life-sciences students specifically. It also gives a perspective about the knowledge and attitudes of the students of life sciences that can be helpful for the experts in the field to gain greater insights. Content validation of the structured questionnaire carried out by an expert is among the strengths of the present study. However, there were certain limitations to the study as well; the questionnaire was not assessed for its test-retest validity and reliability. The findings could not be generalized to the entire community or to the entire student population studying higher degrees considering the study was a single-centric study.

The development of students' knowledge and attitudes about GMOs may be considerably aided by the inclusion of GMO courses in the present curriculum. The results of the present study would be validated by additional research with a focus on students, and their curriculum studies, with larger sample size, in India involving public and private sectors as well. This would enable public and commercial sectors to make more informed policy decisions. Undertaking similar research on the general public or other interest groups and organizations in order to analyze is recommended.
